# Mesenchymal stem cell and endothelial cell interaction restores endothelial permeability via paracrine hepatocyte growth factor *in vitro*

**DOI:** 10.1186/cc14316

**Published:** 2015-03-16

**Authors:** Q Chen, A Liu, H Qiu, Y Yang

**Affiliations:** 1 Southeast University, Nanjing, China

## Introduction

Mesenchymal stem cells (MSCs) have potent stabilizing effects on the vascular endothelium injury, inhibiting endothelial permeability in lung injury via paracrine hepatocyte growth factor (HGF). Recently, it has been indicated that MSCs secreted more factors by MSC-endothelial cell (MSC-EC) interaction. We hypothesized that MSC-EC interaction restored endothelial permeability induced by lipopolysaccharide (LPS) via paracrine HGF.

## Methods

We investigated the endothelial permeability induced by LPS under two co-culture conditions in transwells. HPMECs were added into the upper chambers of cell-culture inserts, while there two different co-culture conditions in the lower side of transwells as follows: MSC-EC interaction group: MSCs and HPMEC contact coculture in the lower chambers; and MSC groups: MSCs only in the lower chambers. The endothelial permeability in the upper side of transwells was detected. Then the concentration of HGF was measured in the culture medium using an enzyme-linked immunosorbent assay kit, following by neutralizing HGF with anti-HGF antibody in the co-culture medium. In addition, VE-cadherin protein expression were measured under the co-culture conditions by western blot, adherens junctions (AJs) protein including F-actin and VE-cadherin were detected by immunofluorescence technique as well.

## Results

The permeability significantly increased after LPS stimulation in a dose-dependent and time-dependent manner (*P *< 0.01). Meanwhile, MSC-EC interaction more significantly decreased endothelial permeability induced by LPS (*P *< 0.05 or *P *< 0.01). Moreover, HGF levels in the MSC-EC interaction group were much higher than those of the MSC group (*P *< 0.01). However, neutralizing HGF with anti-HGF antibody inhibited the role of MSC-EC interaction in improving endothelial permeability (*P *< 0.05). Compared with the MSC group, MSC-EC interaction increased VE-cadherin protein expression (*P *< 0.01), and restored remodeling of F-actin and junctional localization of VEcadherin. However, the MSC effect was significantly blocked by anti-HGF antibody (*P *< 0.05 or *P *< 0.01).

## Conclusion

These data suggest that MSC-EC interaction decreased endothelial permeability induced by LPS, which was mainly attributed to HGF secreted by hMSCs. The main mechanisms of HGF restoring the integrity of EC monolayers are remodeling of endothelial intercellular AJs and decreasing caveolin-1 protein expression.

